# Utilization of two sample *t*-test statistics from redundant probe sets to evaluate different probe set algorithms in GeneChip studies

**DOI:** 10.1186/1471-2105-7-12

**Published:** 2006-01-10

**Authors:** Zihua Hu, Gail R Willsky

**Affiliations:** 1 Center for Computational Research, Department of Biostatistics, Department of Medicine, University at Buffalo, State University of New York (SUNY), Buffalo, NY 14260, USA; 2 Department of Biochemistry, University at Buffalo, State University of New York (SUNY), Buffalo, NY 14214, USA

## Abstract

**Background:**

The choice of probe set algorithms for expression summary in a GeneChip study has a great impact on subsequent gene expression data analysis. Spiked-in cRNAs with known concentration are often used to assess the relative performance of probe set algorithms. Given the fact that the spiked-in cRNAs do not represent endogenously expressed genes in experiments, it becomes increasingly important to have methods to study whether a particular probe set algorithm is more appropriate for a specific dataset, without using such external reference data.

**Results:**

We propose the use of the probe set redundancy feature for evaluating the performance of probe set algorithms, and have presented three approaches for analyzing data variance and result bias using two sample *t*-test statistics from redundant probe sets. These approaches are as follows: 1) analyzing redundant probe set variance based on *t*-statistic rank order, 2) computing correlation of *t*-statistics between redundant probe sets, and 3) analyzing the co-occurrence of replicate redundant probe sets representing differentially expressed genes. We applied these approaches to expression summary data generated from three datasets utilizing individual probe set algorithms of *MAS5.0*, *dChip*, or *RMA*. We also utilized combinations of options from the three probe set algorithms. We found that results from the three approaches were similar within each individual expression summary dataset, and were also in good agreement with previously reported findings by others. We also demonstrate the validity of our findings by independent experimental methods.

**Conclusion:**

All three proposed approaches allowed us to assess the performance of probe set algorithms using the probe set redundancy feature. The analyses of redundant probe set variance based on *t*-statistic rank order and correlation of *t*-statistics between redundant probe sets provide useful tools for data variance analysis, and the co-occurrence of replicate redundant probe sets representing differentially expressed genes allows estimation of result bias. The results also suggest that individual probe set algorithms have dataset-specific performance.

## Background

One of the most promising tools available today to researchers in the life sciences is high-density oligonucleotide array technology [1]. Denoted as GeneChips^®^, high-density oligonucleotide arrays allow one to monitor the relative expression of tens of thousands of genes in a single assay. Upon its introduction within the last decade, GeneChip technology, together with cDNA microarray technology [2], has been viewed as state-of-the-art methodologies that would fundamentally alter the scientific landscape. Supporting this view, the number of published GeneChip studies has exponentially expanded in the past several years to reveal differentially expressed genes (DEGs), gene expression patterns, and genetic networks associated with many experimental conditions [3-5]. Yet the very first step, the generation of expression summary values, on which these studies rely is still open to extensive debate.

On Affymetrix GeneChips, the expression intensity of a gene is reported by a probe set that comprises 11–20 individual probe pairs. Each probe pair contains a perfect match (PM) 25 mer oligonucleotide probe, which is designed to hybridize specifically to a unique gene, and a mismatch (MM) probe of the same length, which differs from the PM probe by one single base in the center of the sequence. The MM probe is intended to measure non-specific hybridization. To compare gene expression levels between GeneChips hybridized with cRNAs of biological interest, the first step is to generate an overall probe set intensity measurement ("expression summary"), which represents the relative expression level of genes from their corresponding probe pairs.

Many probe set algorithms [6-9] have been proposed to generate expression summaries. As each one differs in its strategies for background subtraction, signal intensity normalization between arrays, non-specific hybridization correction, and probe intensity summary, the choice of probe set algorithm for expression summary has a great impact on the subsequent expression data analysis. For example, two lists of DEGs generated from the same dataset by different probe set algorithms were more than 30% different [10]. This discrepancy was also observed in our previous GeneChip studies, in which lists of DEGs from *MAS5.0 *statistical algorithm, Model Based Expression Index (*MBEI *or *dChip*), or Robust Multi-array Analysis (*RMA*) shared similarity between only 10.1% and 36.8% (unpublished observation, Z. Hu and J. F. Collins). Therefore, it is important to compare the relative performance of different probe set algorithms. Comparative studies that have been performed to date are mainly based on spiked-in cRNAs from synthetic datasets [11-14]. These datasets, which contain either small [15] or large [14] numbers of spiked-in cRNAs at various known concentrations, have provided useful means to estimate result bias by comparing either the true expression ratios to observed expression ratios or false discovery rate to true positives, and therefore the relative performance of various probe set algorithms. Despite the important information obtained from these comparative analyses, there is currently no consensus as to which single algorithm yields more reliable results. The performance of individual probe set algorithms might be experiment-specific [16]. Since spiked-in cRNAs do not represent endogenously expressed genes in most experiments, it would be useful to have methods to determine whether a particular probe set algorithm is more appropriate for a specific dataset, without using such external reference data.

In this study, we propose to use probe set redundancy, a quite common feature of Affymetrix GeneChips in which a particular gene is represented by two or more probe sets (denoted as redundant probe sets or RPSs), for the evaluation of the relative performance of different probe set algorithms. We present three approaches for analyzing data variance and result bias using two sample *t*-statistics from redundant probe sets, rather than analyzing them from the hybridization intensities of RPSs on each individual GeneChip. These approaches include 1) RPS variance analysis based on *t*-statistic rank order, 2) correlation analysis of *t*-statistics between RPSs, and 3) analysis of the co-occurrence of replicate RPSs in lists of DEGs. The underling assumption is that these three analysis approaches should result in consistent outcomes; that is, data with lowest RPS variance are expected to also have the strongest RPS correlation and highest RPS co-occurrence in the list of DEGs.

We applied these approaches to expression summary data generated from three datasets utilizing the individual probe set algorithms of *MAS5.0*, *dChip*, or *RMA*. We also utilized combinations of options from the three probe set algorithms [14]. The three datasets we utilized include the GeneLogic dilution dataset [17], a wholly defined control dataset [14], and a dataset from a "real' experimental study on diabetes [18]. The first two datasets have been previously used by others in benchmarking studies to evaluate the performance of probe set algorithms [11-14]. Thus, the comparative results from these previous analyses can be readily used as the standard by which to judge the reliability of the results from our proposed three analysis approaches. Our results demonstrate that probe set redundancy can be used as an internal reference for probe set algorithm evaluation, and therefore provide a novel approach, by which researchers are able to assess the relative performance of different probe set algorithms on their experimental data, without using exogenous controls (*e.g*. spiked-in cRNAs). Furthermore, as GeneChips often contain large numbers of RPSs, our approach allows researchers to better evaluate probe set algorithms, with direct applications to experimental datasets of interest.

## Results

For gene expression studies, the ideal scenario is that RPSs for a given gene will have identical or similar expression values under given experimental conditions. This is however often not the case due to variances introduced by biological variability and differing RPS design, such as alternative RNA splicing [19], probe location within a gene, probe base composition, and location of RPSs on the GeneChip. To reduce or avoid such variances, we propose the use of two sample *t*-statistics of RPSs across all GeneChips in an experiment instead of hybridization intensities of RPSs on each individual GeneChip for analyzing data variance and result bias.

### Data variance and result bias analyses using GeneLogic dilution dataset

#### Data filtering and RPS assignment

In GeneChip studies, a large proportion of genes are usually not expressed across all samples to be compared and, as a common practice, are filtered out before performing statistical analysis. To mimic the real experimental situation, we used *MAS5.0 *"present calls" to filter out probe sets whose expression intensities were close to the background noise across all samples in a *t*-test. We applied the same filtering of at least one "present call" out of 10 samples in a *t*-test to data obtained from each probe set algorithm, since all of them used the same set of CEL files, from which "present calls" were generated. This led to a ~42% data reduction in all comparisons as shown in Table 1. To assign RPSs, we used the UniGene ID (or LocusLink ID) and designated probe sets as redundant if a UniGene ID appeared two or more times in the filtered probe set list. As shown in Table 1, the RPSs constitute ~28% of the total probe sets in all comparisons.

#### RPS variance analysis based on t-statistic rank order

**Figure 1:**
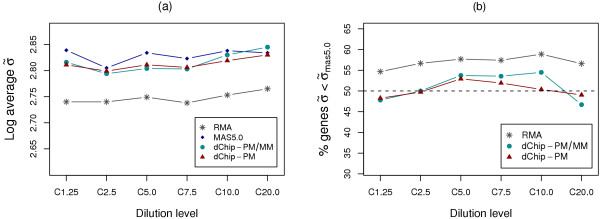
**RPS variance results for GeneLogic dataset**. (a) The log average RPS variances for data from probe set algorithms *RMA*, *MAS5.0*, *dChip-PM*, and *dChip-PM/PM *at all 6 dilution levels (*e.g*. C10.0 stands for dilution level 10.0). (b) The relative RPS variances obtained by comparing RPS variance of individual genes in data from *RMA*, *dChip-PM*, or *dChip-PM/PM *to that of the corresponding genes in data from *MAS5.0*. An above 50% result indicates an overall smaller relative variance than *MAS5.0*.

We first computed RPS variances for individual genes (*e.g*. 1,021 individual genes at the dilution level 7.5) as described in "Methods". We then depicted the overall RPS variances by computing either the average RPS variances from all individual genes or the relative RPS variances for data generated by different probe set algorithms. Data from *RMA *clearly showed much lower variance than those generated by *MAS5.0*, *dChip-PM*, and *dChip-PM/PM *(Figure 1a). This result was highly reproducible at all dilution levels.

To compute relative RPS variance, we used *MAS5.0 *outputs as baselines and compared RPS variance of individual genes to that of the corresponding genes in data from *dChip-PM*, *dChip-PM/MM*, or *RMA*. The relative RPS variance was represented as the percentage of genes whose RPS variance was smaller than the corresponding genes in data generated by *MAS5.0*. Thus, a number greater than 50% indicated an overall smaller relative RPS variance than *MAS5.0*. In agreement with the average RPS variance, data from *RMA *displayed smaller relative RPS variances when compared to those from other probe set algorithms (Figure 1b), in which 54.7% to 58.9% of genes had smaller RPS variance than those from *MAS5.0*. By contrast, the difference in the relative RPS variance between data generated by *MAS5.0 *and those from *dChip-PM*, or *dChip-PM/MM *was not consistent at different dilution levels, in which 46.7% to 54.4% of genes had smaller RPS variance than those from *MAS5.0*. We also verified the statistical significance of the relative RPS variance between *MAS5.0 *and *RMA*, *dChip-PM*, or *dChip-PM/MM *by using the Wilcoxon signed-rank test. We found that median RPS variances for data from *RMA *were significantly different from those from *MAS5.0*, with < 10^-5 ^*p*-values for all dilution levels. Conversely, data from both *dChip-PM *and *dChip-PM/MM *did not display significant differences in RPS variance for all dilution levels, with the exception being dilution level 7.5 in which result from *dChip-PM *had *p*-value of 0.031. Thus, of all data generated by different probe set algorithms, those from *RMA *had the smallest RPS variance.

#### Correlation analysis of t-statistics between RPSs

**Figure 2:**
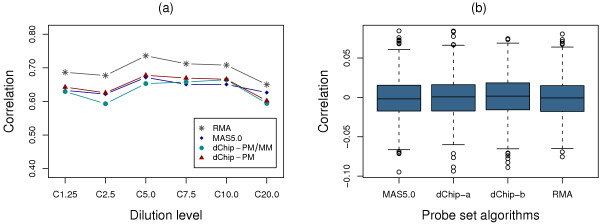
**Correlation of RPSs for GeneLogic dataset**. (a) Correlation of *t*-test statistics between RPSs for data from probe set algorithm *RMA*, *MAS5.0*, *dChip-PM*, and *dChip-PM/PM *at all 6 dilution levels (*e.g*. C10.0 stands for dilution level 10.0). (b) The distributions of the correlation coefficients from 1,000 simulated datasets with random *t*-test statistic pairs for data from each probe set algorithm. The results from dilution level 7.5 are shown. Correlation coefficients for all data from *RMA*, *MAS5.0*, *dChip-PM *(dChip-a) and *dChip-PM/PM *(dChip-b) are centered at zero.

The second approach we used for analyzing data variance was to investigate the correlation of *t*-statistics between RPSs. The underling hypothesis is that *t*-statistics of RPSs with similar expression patterns in two sample groups should be highly correlated. In this study, we employed Pearson correlation coefficients to estimate the correlation between paired *t*-statistics from RPSs. For genes with more than two RPSs, we generated all combinations of paired *t*-statistics to the number of (N2)
 MathType@MTEF@5@5@+=feaafiart1ev1aaatCvAUfKttLearuWrP9MDH5MBPbIqV92AaeXatLxBI9gBaebbnrfifHhDYfgasaacH8akY=wiFfYdH8Gipec8Eeeu0xXdbba9frFj0=OqFfea0dXdd9vqai=hGuQ8kuc9pgc9s8qqaq=dirpe0xb9q8qiLsFr0=vr0=vr0dc8meaabaqaciaacaGaaeqabaqabeGadaaakeaadaqadaabaeqabaGaemOta4eabaGaeGOmaidaaiaawIcacaGLPaaaaaa@3053@ pairs, where *N *is the number of RPSs for a given gene. As shown in Figure 2a, the paired *t*-statistics clearly show stronger correlations for data generated by *RMA *than those from other probe set algorithms at all dilution levels, indicating that data from *RMA *have more similar RPS expression patterns in two sample groups, and thus possibly lower variance.

We were concerned that the superior results from data generated by *RMA *might be associated with stronger random or biological correlation, which could potentially affect the outcomes in the comparisons described above. To investigate this possibility, we performed further analysis to estimate the correlation for lists of randomly paired *t*-statistics. These *t*-statistics were randomly sampled without replacement from *t*-statistics of all probe sets in the corresponding expression summary datasets and then used to form *t*-statistic pairs of the same size as those used in the correlation analysis for RPSs. As an example, one of the random correlation comparisons, dilution level 7.5, is shown in Figure 2b. The distributions of the correlation coefficients from 1,000 simulated lists of randomly paired *t*-statistics are centered at zero for each probe set algorithm, suggesting that no random or biological correlation exists in any of the data used for RPS correlation analysis. Similar results were also obtained from the other 5 dilution levels (data not shown).

#### Analysis of co-occurrence of replicate RPSs in lists of DEGs

We define "replicate RPSs" as those with two or more RPSs for a given gene in the final list of DEGs, and those with only one RPS as "single RPS". In the ideal scenario of no RPS variance, we expect an "all-or-none" expression pattern of RPSs for individual genes in the list of DEGs. Therefore, the degree of deviation from this ideal scenario provides a tool for researchers to estimate the result bias. The approach we used was to calculate the proportion of replicate RPSs out of both replicate and single RPSs in DEGs.

**Figure 3:**
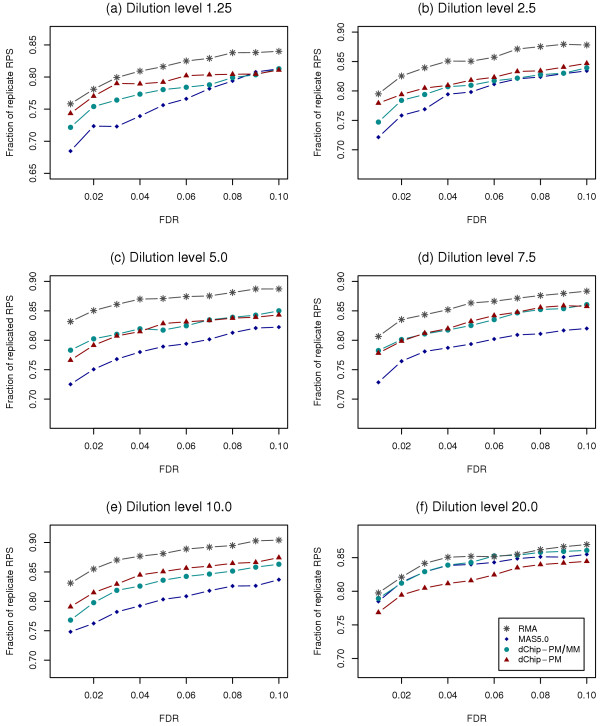
**Co-occurrence rate of replicate RPSs in DEGs for GeneLogic dataset**. The fraction of replicate RPSs out of all RPSs in DEG list for data from probe set algorithm *RMA*, *MAS5.0*, *dChip-PM*, and *dChip-PM/PM *is shown at individual FDR cutoffs. Comparison results for 6 dilution levels are shown separately: (a) for dilution level 1.25, (b) for dilution level 2.5, (c) for dilution level 5.0, (d) for dilution level 7.5, (e) for dilution level 10.0, and (f) for dilution level 20.0

To estimate the fraction of replicate RPS in the list of DEGs, we first used the Benjamini-Hochberg [20] approach to control experiment-wise false discovery rate (FDR) to select significant genes. The adjustment for the multiple testing allowed us to determine significant *p*-values and thus establish statistical cutoffs for significant genes. The relative proportions of replicate RPSs out of all RPSs in DEG list from different probe set algorithms were consistent in all dilution levels (Figure 3a–3f), when using the FDR cutoffs in a range similar to what would be utilized in actual experimental situations (0.01 to 0.1). Whereas results for *RMA *displayed the highest co-occurrence rate, results for *MAS5.0 *showed the lowest co-occurrence rates. Results for *dChip-PM *and *dChip-PM/MM *had similar co-occurrence rates, which were between those from *RMA *and *MAS5.0*. These results were highly reproducible at all dilution levels, except dilution level 20.0 from *MAS5.0*.

To investigate whether data filtering would affect the outcomes of RPS variance and result bias analysis, we also utilized the entire dataset, in which the RPSs constitute 34.9% of the total probe sets, to perform the analyses. Results of RPS variance, correlation of *t*-statistics between RPSs, and the co-occurrence of replicate RPSs in DEGs all strongly agreed with results obtained from data filtered by one "present call", but with relatively smaller differences between data from *RMA *and data from other probe set algorithms (data not shown). These results suggest that data filtering may improve the performance of the three proposed approaches, although data can be directly used without filtering for RPS variance and result bias analysis.

Taken together, results from all three approaches indicated that data generated by *RMA *were superior to those generated by other probe set algorithms. This fact was reflected by RPSs with lower variance, stronger correlation, and higher co-occurrence rates in final DEG list. These results were also in agreement with previous benchmarking studies using the GeneLogic dilution dataset [11-13], which indicated that *RMA *had a superior performance over other probe set algorithms, as demonstrated by the lower expression variance and smaller result bias. Although the performance of *RMA *is better than that of other probe set algorithms we tested, it is worthy to note that *RMA *is not necessarily the most robust probe set algorithm in most cases, which was illustrated by the relative improvement of RPS variances and the findings from the remainder of this study using the two other datasets.

### Data variance and result bias analyses using the wholly defined control dataset

#### Summary of results from previous wholly control dataset study

This wholly defined control dataset was constructed and has been used by Choe *et al*. [14] to evaluate the methods and analysis options for Affymetrix GeneChips to identify DEGs. These options, which were derived from several probe set algorithms commonly used for GeneChip analysis, include background correction, PM correction, probe level normalization, expression summary, and probe set level normalization. These authors have applied all possible combinations of options to the data to assess whether some steps are more critical than others in maximizing the detection of DEGs. Using receiver operator characteristic (ROC) curves of false discovery rate vs. true positives, they have facilitated the assessment of the performance of various options and have reported the following findings:

**1. **A second loess normalization at the probe set level yielded a superior result.

**2. **Among various robust estimators used for expression summary, the *median polish *method performed the best.

**3. **Among different PM correction options, the method in *MAS5.0 *had a superior performance over the others.

**4. **Among the background correction methods, the *MAS5.0 *method generally performed better than the *RMA *method.

**5. **No clearly superior normalization method was found at the probe level.

**6. **Ten best expression summary datasets, which maximized the detection of DEGs and minimized false positives, were generated by a combination of optimal options.

These findings, which resulted from the analyses of a large number of expression summary datasets generated from the wholly control dataset by different combinations of options, provide standards to which our current assessments can be compared.

#### Comparison of results from the three proposed approaches and previous studies

We made use of RPSs from the 3,860 individual cRNAs for the study reported here, as they are known sequences, and the complete annotations can be found in the Drosophila Gene Collection release 1.0 [21]. Based on Drosophila Gene Collection clone IDs, we were able to obtain 582 RPSs, which represented 268 unique genes, from the 3,860 cRNAs. We computed data variance at 8 levels with fold change equal to or greater than 1.0, 1.2, 1.5, 1.7, 2.0, 2.5, 3.0, 3.5, or 4.0, although the majority of the RPSs (69.2%) were spiked in at identical concentrations between the two sample groups.

**Figure 4:**
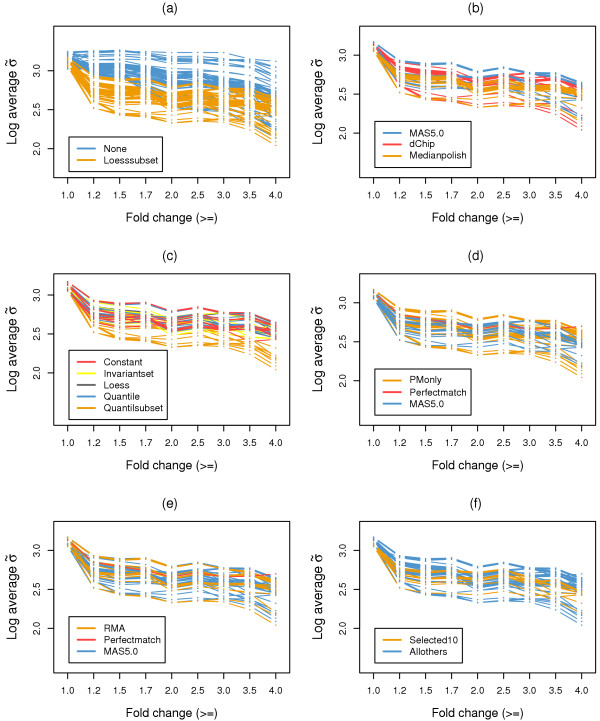
**RPS variance results for the wholly defined control dataset**. The log average RPS variances for expression summary datasets generated from combinations of options from different probe set algorithms. RPS variances were computed at 8 levels with fold change equal to or greater than 1.0, 1.2, 1.5, 1.7, 2.0, 2.5, 3.0, 3.5, or 4.0 between two test sample groups. (a) All 110 expression summary datasets for the comparison of normalization options at the probe set level (b-f). To match the analysis from Choe *et al*. [14], only 55 expression summary datasets involving the second normalization step are shown. (b) Comparison of expression summary options. (c) Comparison of normalization options. (d) Comparison of PM correction options. (e) Comparison of background correction options. (f) Comparison of the selected 10 expression summary datasets to all others.

**Figure 5:**
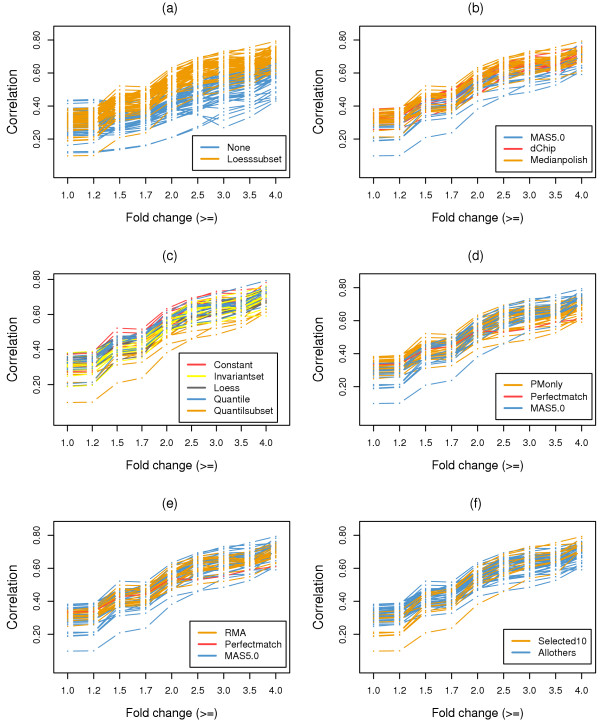
**Correlation of RPSs results for the wholly defined control dataset**. Correlation of *t*-test statistics between RPSs for expression summary datasets generated from combinations of options utilizing different probe set algorithms. The same analysis procedures as in Figure 4 were applied. (a) Comparison of normalization options at the probe set level. (b) Comparison of expression summary options. (c) Comparison of normalization options. (d) Comparison of PM correction options. (e) Comparison of background correction options. (f) Comparison of the selected 10 expression summary datasets to all others.

**Figure 6:**
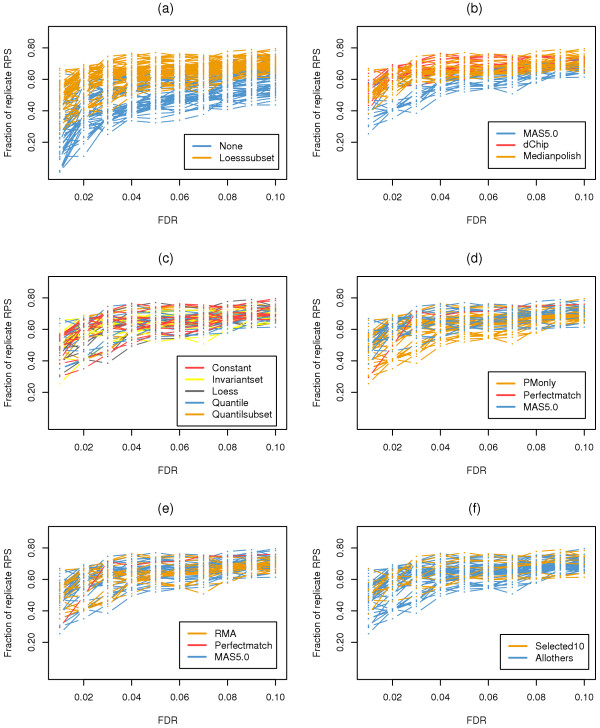
**Co-occurrence rate of replicate RPSs in DEGs for the wholly defined control dataset**. The fraction of replicate RPSs out of all RPSs in DEGs for expression summary datasets generated utilizing combinations of options from different probe set algorithms. The same analysis procedures as in Figure 4 were applied. (a) Comparison of normalization options at the probe set level. (b) Comparison of expression summary options. (c) Comparison of normalization options. (d) Comparison of PM correction options. (e) Comparison of background correction options. (f) Comparison of the selected 10 expression summary datasets to all others.

We employed the same analysis procedures as described for the GeneLogic dilution dataset to compute variance, correlation, and co-occurrence for RPSs. To make our results comparable to those from Choe *et al*. [14], we used 110 expression summary datasets to perform the comparison of options for probe set level normalization, and used 55 of the expression summary datasets that were generated with the loess normalization option (orange lines in Figure 4a, Figure 5a, and Figure 6a) for comparison of the remaining options.

Among our three proposed analysis approaches, results of RPS variances (Figure 4) were consistent with findings from Choe *et al*. [14]. First, a second loess normalization at the probe set level consistently decreased the RPS variance, as can be seen from the log average RPS variance across all fold change cutoffs in Figure 4a. This is also true for expression summary options, for which results from the *median polish *method displayed relatively lower RPS variance (Figure 4b). Second, Figure 4d clearly show that expression summary datasets from *MAS5.0 *PM correction have lower RPS variances when all RPSs are considered (fold change >= 1.0), which is similar to previous study [14]. Similar results were also obtained for the 10 best expression summary datasets (Figure 4f), which displayed relatively lower RPS variance compared to the remaining expression summary datasets. It is noteworthy that in the higher fold change ranges (fold change >= 1.2 – 4.0) the difference in RPS variance is not apparent between different PM correction options, which is also true for the 10 best expression summary datasets as compared to all other expression summary datasets. This may be due to the smaller number of RPSs used when computing RPS variance. For the remaining two comparisons, with data normalization at the probe level (Figure 4c) and background correction (Figure 4e), no significant differences was observed between options.

In agreement with the findings reported by Choe *et al*. [14], our additional analyses indicated that a second loess normalization at the probe set level substantially increased both correlation (Figure 5a) and co-occurrence (Figure 6a) of RPSs. Additionally, the *median polish *method for expression summary had a much better performance than MAS5.0, as can be seen from the higher correlations shown in Figure 5b as well as higher co-occurrence of RPSs shown in Figure 6b. For the remaining options, no single one stood out as clearly superior, but some options generally performed better than others. For example, the co-occurrences of RPSs from the *MAS5.0 *option for PM correction scored higher (Figure 6d), demonstrating its relatively better performance. This was also true for the 10 best expression summary datasets (Figure 6f).

### Data variance and result bias analyses using our experimental diabetes dataset

#### Dataset features and analysis results

The third dataset came from an experimental dataset designed to study diabetes. To increase the sensitivity for detecting DEGs, we developed a statistical approach to eliminate expression outliers across biological replicates. Briefly, for each individual probe set from 5 biological replicates one of them, whose expression value had the largest deviation from the sample mean x¯=15∑i=15xi
 MathType@MTEF@5@5@+=feaafiart1ev1aaatCvAUfKttLearuWrP9MDH5MBPbIqV92AaeXatLxBI9gBaebbnrfifHhDYfgasaacH8akY=wiFfYdH8Gipec8Eeeu0xXdbba9frFj0=OqFfea0dXdd9vqai=hGuQ8kuc9pgc9s8qqaq=dirpe0xb9q8qiLsFr0=vr0=vr0dc8meaabaqaciaacaGaaeqabaqabeGadaaakeaacuWG4baEgaqeaiabg2da9maalaaabaGaeGymaedabaGaeGynaudaamaaqadabaGaemiEaG3aaSbaaSqaaiabdMgaPbqabaaabaGaemyAaKMaeyypa0JaeGymaedabaGaeGynaudaniabggHiLdaaaa@3A7B@, was first selected as the putative outlier *O*_*p*_. The sample mean x¯(i)=14∑i′=1i′≠i5xi
 MathType@MTEF@5@5@+=feaafiart1ev1aaatCvAUfKttLearuWrP9MDH5MBPbIqV92AaeXatLxBI9gBaebbnrfifHhDYfgasaacH8akY=wiFfYdH8Gipec8Eeeu0xXdbba9frFj0=OqFfea0dXdd9vqai=hGuQ8kuc9pgc9s8qqaq=dirpe0xb9q8qiLsFr0=vr0=vr0dc8meaabaqaciaacaGaaeqabaqabeGadaaakeaacuWG4baEgaqeamaaBaaaleaacqGGOaakcqWGPbqAcqGGPaqkaeqaaOGaeyypa0ZaaSaaaeaacqaIXaqmaeaacqaI0aanaaWaaabCaeaacqWG4baEdaWgaaWcbaGaemyAaKgabeaaaqaabeqaaiqbdMgaPzaafaGaeyypa0JaeGymaedabaGafmyAaKMbauaacqGHGjsUcqWGPbqAaaqaaiabiwda1aqdcqGHris5aaaa@4298@ and standard deviation *s*_(*i*)_(*x*_*i*_) from the rest 4 replicates were subsequently recomputed, which was used to build a 99% confidence interval x¯(i)±tsx¯(i)
 MathType@MTEF@5@5@+=feaafiart1ev1aaatCvAUfKttLearuWrP9MDH5MBPbIqV92AaeXatLxBI9gBaebbnrfifHhDYfgasaacH8akY=wiFfYdH8Gipec8Eeeu0xXdbba9frFj0=OqFfea0dXdd9vqai=hGuQ8kuc9pgc9s8qqaq=dirpe0xb9q8qiLsFr0=vr0=vr0dc8meaabaqaciaacaGaaeqabaqabeGadaaakeaacuWG4baEgaqeamaaBaaaleaacqGGOaakcqWGPbqAcqGGPaqkaeqaaOGaeyySaeRaemiDaqNaem4Cam3aaSbaaSqaaiqbdIha4zaaraWaaSbaaWqaaiabcIcaOiabdMgaPjabcMcaPaqabaaaleqaaaaa@3B50@ using the *t *distribution, where sx¯(i)=s(i)(xi)/4
 MathType@MTEF@5@5@+=feaafiart1ev1aaatCvAUfKttLearuWrP9MDH5MBPbIqV92AaeXatLxBI9gBaebbnrfifHhDYfgasaacH8akY=wiFfYdH8Gipec8Eeeu0xXdbba9frFj0=OqFfea0dXdd9vqai=hGuQ8kuc9pgc9s8qqaq=dirpe0xb9q8qiLsFr0=vr0=vr0dc8meaabaqaciaacaGaaeqabaqabeGadaaakeaacqWGZbWCdaWgaaWcbaGafmiEaGNbaebadaWgaaadbaGaeiikaGIaemyAaKMaeiykaKcabeaaaSqabaGccqGH9aqpcqWGZbWCdaWgaaWcbaGaeiikaGIaemyAaKMaeiykaKcabeaakiabcIcaOiabdIha4naaBaaaleaacqWGPbqAaeqaaOGaeiykaKIaei4la8YaaOaaaeaacqaI0aanaSqabaaaaa@3F92@ and t = 5.84. The sample mean x¯(i)
 MathType@MTEF@5@5@+=feaafiart1ev1aaatCvAUfKttLearuWrP9MDH5MBPbIqV92AaeXatLxBI9gBaebbnrfifHhDYfgasaacH8akY=wiFfYdH8Gipec8Eeeu0xXdbba9frFj0=OqFfea0dXdd9vqai=hGuQ8kuc9pgc9s8qqaq=dirpe0xb9q8qiLsFr0=vr0=vr0dc8meaabaqaciaacaGaaeqabaqabeGadaaakeaacuWG4baEgaqeamaaBaaaleaacqGGOaakcqWGPbqAcqGGPaqkaeqaaaaa@3176@ was then used to replace the outlier as follow:

Op={x¯(i)if Op>x˙(i)+tsx¯(i)or Op<x˙(i)−tsx¯(i)Opotherwise
 MathType@MTEF@5@5@+=feaafiart1ev1aaatCvAUfKttLearuWrP9MDH5MBPbIqV92AaeXatLxBI9gBaebbnrfifHhDYfgasaacH8akY=wiFfYdH8Gipec8Eeeu0xXdbba9frFj0=OqFfea0dXdd9vqai=hGuQ8kuc9pgc9s8qqaq=dirpe0xb9q8qiLsFr0=vr0=vr0dc8meaabaqaciaacaGaaeqabaqabeGadaaakeaacqWGpbWtdaWgaaWcbaGaemiCaahabeaakiabg2da9maaceaabaqbaeaabiWaaaqaaiqbdIha4zaaraWaaSbaaSqaaiabcIcaOiabdMgaPjabcMcaPaqabaaakeaacqqGPbqAcqqGMbGzcaaMc8Uaem4ta80aaSbaaSqaaiabdchaWbqabaGccqGH+aGpcuWG4baEgaGaamaaBaaaleaacqGGOaakcqWGPbqAcqGGPaqkaeqaaOGaey4kaSIaemiDaqNaem4Cam3aaSbaaSqaaiqbdIha4zaaraWaaSbaaWqaaiabcIcaOiabdMgaPjabcMcaPaqabaaaleqaaaGcbaGaee4Ba8MaeeOCaiNaaGPaVlabd+eapnaaBaaaleaacqWGWbaCaeqaaOGaeyipaWJafmiEaGNbaiaadaWgaaWcbaGaeiikaGIaemyAaKMaeiykaKcabeaakiabgkHiTiabdsha0jabdohaZnaaBaaaleaacuWG4baEgaqeamaaBaaameaacqGGOaakcqWGPbqAcqGGPaqkaeqaaaWcbeaaaOqaaiabd+eapnaaBaaaleaacqWGWbaCaeqaaaGcbaGaee4Ba8MaeeiDaqNaeeiAaGMaeeyzauMaeeOCaiNaee4DaCNaeeyAaKMaee4CamNaeeyzaugabaaaaaGaay5Eaaaaaa@710C@

This approach improves the detection sensitivity only for the probe sets that have high homogeneity in expression intensity across at least four out of five biological replicates, and therefore genes identified as significant have high rate of accuracy. This dataset, in which the RPSs constitute 34.6% of the total probe sets after performing data filtering, provided an example of a typical "real" experiment, from which biological and experimental validation was readily available.

**Figure 7:**
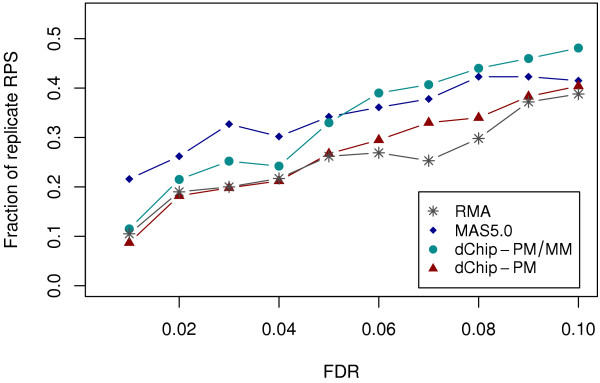
**Co-occurrence rate of replicate RPSs in DEGs for the diabetes dataset**. The fraction of replicate RPSs out of all RPSs in DEGs for data from probe set algorithm *RMA*, *MAS5.0*, *dChip-PM*, and *dChip-PM/PM *is shown at individual FDR cutoffs.

We followed the same analysis procedures as used for the GeneLogic dilution and wholly defined control datasets, and found that results from our 3 proposed approaches were generally in agreement. Data generated by *MAS5.0 *and *dChip-PM/MM *were superior as compared to data from *RMA *and *dChip-PM*. This was especially apparent from the analysis of co-occurrence of replicate RPSs in list of DEGs, in which co-occurrence rates for data from *MAS5.0 *and *dChip-PM/MM *were higher than data from *RMA *and *dChip-PM *(Figure 7), when controlling the FDR in the range used in actual experimental situations (0.01 to 0.1). In support of this finding, RPS variance analysisalso indicated that data from *MAS5.0 *and *dChip-PM/MM *had smaller variance than data from the other 2 probe set algorithms. For example, for the relative RPS variance analysis the percentage of genes whose RPS variance was smaller than the corresponding genes from *RMA *was 52.5%, 52.4%, and 50.1% for *MAS5.0*, *dChip-PM/MM*, and *DChip-PM*, respectively.

#### Quantitative RT-PCR validation

**Figure 8:**
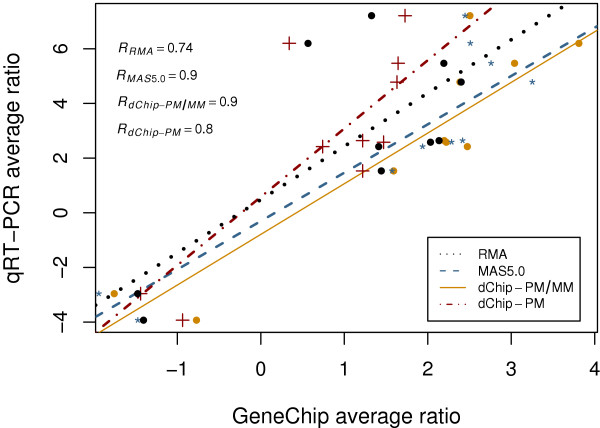
**Correlation of qRT-PCR fold changes with those from GeneChip study**. For 10 genes, qRT-PCR average ratio from biological replicates between diabetic and normal animals vs. the average ratio in GeneChips from different probe set algorithms are depicted as color-coded symbols, and linear fittings for the data are depicted as color-coded lines. The correlation coefficients comparing data from each probe set algorithm to those from qRT-PCR are shown in the upper left corner.

Quantitative RT-PCR (qRT-PCR) is a common and useful method for confirming DEGs, and thus for validating results from GeneChip experiments. Ten genes distributed among different functional groups identified in a previous study were selected for qRT-PCR studies [18]. In that study data in diabetic rat group was compared to all those in the normal rat group as suggested by Affymetrix [22], which involved looking at a 5 × 5 matrix for the experiment. The specific genes, primers used, and fold change values found by PCR are shown in Table 3. For each of the 10 genes, qRT-PCR average fold change from biological replicates between diabetic and control animals were first computed and then compared with the average fold change from GeneChips. As shown in Figure 8, the fold changes from *MAS5.0 *and *dChip-PM/MM *are highly correlated with those from qRT-PCR, with correlation coefficients of 0.9 for both methods. Conversely, the fold changes from *RMA *and *dChip-PM *showed relatively weak correlation with those from qRT-PCR, with correlation coefficients of 0.8 for *dChip-PM *and 0.74 for *RMA*.

#### Biological validation

We also explored the level of concordance of the biological themes for DEGs to the results of data variance and result bias analyses. Taking advantage of known knowledge from previous diabetes studies, we categorized the statistically over-represented biological process categories for DEGs by DAVID 2.0 [23]. Because diabetes is a multifactorial disease [24], which leads to substantial changes in gene expression in a broad range of biological function categories, the number of over-represented biological processes is usually overwhelming, with many overlaps found using different probe set algorithms. In this study, the enriched functional categories included protein biosynthesis, macromolecule metabolism, physiological process, ribosome biogenesis, and regulation of cell proliferation. Nevertheless, we determined uniquely over-represented biological process from DEG lists generated by the chosen probe set algorithms to determine which one was superior from a physiological perspective.

Previous studies using GenChips with samples obtained from mice with streptozotocin-induced diabetes indicated that major diabetes related changes in gene expression included carbohydrate and lipid metabolism, energy metabolism, cellular transport and vesicle trafficking, intracellular signaling, and response to stress [25]. In our GeneChip studies reported here RNA was obtained from normal rats and rats with streptozotocin-induced diabetes. As shown in Table 2, biological function categories associated with diabetes [24,25] such as intracellular signaling cascade, lipoprotein biosynthesis, steroid metabolism, negative regulation of transcription, phosphagen biosynthesis, and response to stress are enriched to a larger extent in DEGs either from *MAS5.0 *or *dChip-PM/MM*. On the other hand, DEGs from *dChip-PM *have only one diabetes-related biological process, and all uniquely enriched biological processes for DEGs from *RMA *do not appear to be directly related to the diabetes phenotype. Thus, DEG lists generated by *MAS5.0 *and *dChip-PM/MM *were most relevant to the disease state being studied.

## Discussion

The choice of probe set algorithms used for expression summary in GeneChip studies has a major impact on differential gene expression analysis, as important differences exist in the way the expression summary is generated using the various algorithms. Spiked-in cRNAs with known concentrations are often used to assess the relative performance of different probe set algorithms. However, this approach has apparent limitations because spiked-in cRNAs are not endogenously expressed genes in experimental systems of biological interest. In addition, if we assume that the performance of individual probe set algorithms is experiment-specific [16], the lack of spiked-in cRNA controls in most experiments prevents this approach from applying to many datasets. In contrast, our proposed new approach of using probe set redundancy addresses this issue adequately, as GeneChips often contain large numbers of RPSs, which represent experimental genes under study.

For comparison purposes, we initially employed the GeneLogic dilution dataset to directly estimate RPS variances using the expression values of RPSs by the method of Barash *et al *[10]. Notably, although RPS variances based on expression values differed significantly among data from different probe set algorithms (data not shown), the results did not agree with both ours and those previously reported by others [11-13], indicating that the estimation of RPS variance was less reliable using expression values than using *t*-statistic values.

For our study, rather than analyzing data variance and result bias from the hybridization intensities of RPSs on each individual GeneChip [10], we chose to use two sample *t*-test statistics to compare hybridization intensities of individual RPSs across all GeneChips in an experiment. This approach allowed us to obviate the variance introduced by differing probe set design and location of the probe within a primary or alternatively spliced transcript, which introduces RPS variance on an individual GeneChip. Other important issues are biological variability between samples, technical issues related to RNA integrity and sample preparation, and non-specific, cross-reactivity of probes [26]. If we thus assume that these introduced variances have the same impact on results obtained by different probe set algorithms (as they all use the same dataset), then we can minimize RPS variance on an individual GeneChip by comparing expression between different GeneChips and can thus effectively evaluate the performance of different probe set algorithms. The resulting *t*-statistics used to analyze RPSs across GeneChips would therefore reflect whether or not the hybridization intensity differences of RPSs within each sample group were consistent with the differences in other sample groups.

The validity of using RPS and two sample *t*-test statistics for the evaluation of probe set algorithm performance was clearly demonstrated by the correlation analysis. For example, in the GeneLogic dilution dataset, strong correlations (0.59 to 0.74) of *t*-statistics between RPSs were obtained from data for all probe set algorithms (Figure 2a), while no correlation existed for randomly paired *t*-statistics from corresponding expression summary datasets (Figure 2b). It is noteworthy that correlations of RPS *t*-statistics were less than the ideal situation of 1 (Figure 2a), which might be due to the variances introduced from such confounding factors as cross-hybridization and recognition of alternatively spliced transcripts as discussed above and suggested by others [26]. Nevertheless, the use of RPS and two sample *t*-test statistics for the evaluation of different probe set algorithm performance is clearly appropriate, as the RPSs usually constitute a large portion of the probe sets on GeneChips and the introduced variances have the same impact on results obtained by different probe set algorithms.

We utilized 3 approaches, RPS variance analysis, correlation analysis of *t*-statistics between RPSs, and analysis of the co-occurrence of replicate RPSs in DEGs, as tools to judge data variances and result bias. We also assumed that results from the three approaches would be consistent for the same expression summary dataset. Out of the three approaches, however, the correlation analysis proved to be the least useful, as correlation measures the trend of *t*-statistics instead of absolute variances. This was the case from the results of the wholly defined control dataset, in which the correlation analysis displayed the lowest level of concordance to the results from Choe *et al*. [14]. While both co-occurrence of replicate RPS analyses and RPS variance are more robust than correlation analysis, the analysis of co-occurrence of replicate RPSs in DEGs provides an approach for researchers to estimate result bias, and RPS variance analysis provides a tool for data variance analysis.

Three datasets used in this study come from different sources and had differing data quality and features, they therefore nicely represent most situations. Above all, results of data variance and result bias for expression summary datasets, whether they were generated from individual probe set algorithms or combinations of options from a few probe set algorithms, agreed well with each other in each individual test dataset and were also in good agreement with either previous findings by others or experimental evaluation from this study. First, RPS variance analyses using expression summary datasets generated from the GeneLogic dilution dataset by individual probe set algorithms indicated that data from *RMA *had an overall better quality, which was in good agreement with findings by others [11-13]. Second, our results from the wholly defined control dataset agreed with those conclusions from Choe *et al*. [14]. However, unlike data generated from GeneLogic dilution dataset, the expression summary datasets were generated by combinations of options from a few probe set algorithms and were in large numbers, thus allowing more sophisticated and comprehensive assessments. Finally, for our diabetes dataset, results from both experimental validation by qRT-PCR and biological validation by functional classification for DEGs agreed well with results from data variance and bias analyses, in which data from probe set algorithms using both PM and MM probes for expression summary displayed lower variance and bias.

Although our proposed analysis approaches were to evaluate the relative probe set algorithm performance within individual datasets, it was interesting to note that the fraction of replicate RPS for DEGs in the diabetes dataset was smaller than those in the other two datasets. This difference was also observed between unfiltered (data not shown) and one "present call" filtered GeneLogic dilution dataset, in which fractions of replicate RPSs for DEGs in the one "present call" filtered data were 5–11% higher than those obtained from data without filtering. These differences could be due to the different nature of RPSs on the different GeneChips or differing data quality.

It is noteworthy that varying conclusions for probe set algorithm performance were drawn by using different datasets. Irizarry *et al*. [12] found that *RMA *performed best among a few probe set algorithms tested, when using the GeneLogic dilution dataset. On the other hand, using the wholly defined control datasets no best single method was found by Choe *et al*. [14], who instead suggested a best-route combinations of analysis options from *MAS5.0*, *RMA*, *dChip*, and an additional loess normalization at the probe set level. Results from the diabetes dataset used in this study, when used for the evaluation of probe set algorithm performance, indicated that probe set algorithms using both PM and MM probe sets for expression summary gave better results than those methods using only PM for expression summary, such as *RMA*. Overall these results suggest that individual probe set algorithms may have experiment-specific performance. Moreover, the strong correlation between the results from previous probe set algorithm assessments and those from this study demonstrate that our proposed novel approaches based on RPS analysis are not dependent upon individual probe set algorithm performance, and are thus very likely to be reliable and reproducible.

## Conclusion

An important issue in the analysis of gene expression data from Affymetrix GeneChips is to choose a probe set algorithm for expression summary. In this study, we have proposed the use of probe set redundancy to evaluate the performance of different probe set algorithms, and have presented three approaches for assessing data variance and result bias using two sample *t*-test statistics. These methods include RPS variance analysis based on *t*-statistic rank order, correlation analysis between *t*-statistics of RPSs, and analysis of co-occurrence of replicate RPSs in DEG list. The main advantage of our approaches lies in the fact that we do not make use of external reference data, but rather investigate data variance and result bias based on RPSs, which often constitute a large portion of and have direct relevance to the genes under study. Furthermore, the use of *t*-statistics allows us to reduce or avoid RPS variances introduced from both biological variability and differing probe design. To assess the usefulness of the three proposed approaches, we have applied them to three diverse datasets using a few widely used probe set algorithms. Results from all three approaches not only agreed well with each other in each individual test dataset but they were in good agreement with either previous findings by others or experimental validation from this study. These approaches provide an alternative method to determine data variance and result bias, without the use of exogenous controls, and are thus useful for the assessment of probe set algorithm performance. The results also suggest that individual probe set algorithms have dataset-specific performance.

## Methods

### Datasets

#### GeneLogic dilution/mixture dataset

The GeneLogic dilution/mixture dataset [17] comprises 75 HG-U95A GeneChips to which two sources of RNA, human liver tissue and a central nervous system cell line (CNS), have been hybridized in various dilutions and combinations. Of these 75 GeneChips, 60 of them were hybridized with cRNA from either liver tissue or CNS at the concentrations of 1.25, 2.5, 5.0, 7.5, 10.0 or 20.0 μg, with 5 replicate GeneChips for each dilution level. Data from these 60 GeneChips, called the GeneLogic dilution dataset, have been used in this study.

#### A wholly defined control dataset

Choe *et al*. [14] recently performed a study to evaluate the methods and analysis options for Affymetrix GeneChips for identifying DEGs. They have constructed a wholly defined control dataset to mimic the scenario of comparing two samples in a microarray experiment. This dataset comprises 3,860 individual *Drosophila *cRNAs of known sequences. Out of the 3,860 cRNAs, 1309 have been spiked in with different ratios of 1.2, 1.5, 1.7, 2.0, 2.5, 3.0, 3.5, or 4.0 between two test sample groups, and the remaining 2,551 cRNAs have the same relative concentration in each test sample group. Each sample has hybridized in triplicate to Affymetrix *Drosophila *arrays (DrosGenome1).

#### Diabetes dataset

The third dataset consists of 10 Affymetrix GeneChips from diabetes study [18]. Gene expression data was obtained from 2 groups of rats: normal rats and rats with streptozotocin-induced diabetes for four weeks. In each group of 5 animals, labeled cRNA was prepared from 10.0 μg total RNA obtained from the leg muscle of one animal and was separately hybridized to one Affymetrix U34A Rat GeneChip. GeneChips were scanned at the Gene Expression Core Facility at Roswell Park Cancer Institute.

### Generating expression summary values

Out of many probe set algorithms, the most widely used are the *MAS5.0 *statistical algorithm from Affymetrix [6], the Model Based Expression (*dChip*) Index of Li and Wong [7,8], and the Robust Multi-Chip Analysis (*RMA*) of Irizarry *et al*. [9]. All of them are based on the statistical model of gene expression values as a function of the probe level intensities. There are, however, important differences between these probe set algorithms.

*MAS5.0 *is based on the assumption of homogeneity of probe affinity and weights each probe in a probe set equally. *MAS5.0 *uses both PM and MM probes for expression summary from the average value of PM – MM in a probe set and employs the Tukey's Biweight weighted average of the probe level signals to avoid sensitivity to outlier probe intensity. Unlike *dChip *and *RMA*, *MAS5.0 *summarizes expression individually for each GeneChip and performs a scaling normalization at the level of expression summary.

The *dChip *method assumes different probe affinity in a probe set and considers a probe value as the product of gene expression and probe-sensitivity index. The *dChip *method uses a multiplicative model and employs probe values of the same probe set from all GeneChips in an experiment to iteratively estimate gene expression-specific index as well as probe affinity-specific index for expression summary. Normalization in *dChip *is carried out at the probe level, which makes use of an invariant subset of probes that have small within-subset rank differences between GeneChips. Additionally, the *dChip *performs the expression measures using either only the PM probes ("*dChip-PM"*) or both PM and MM probes ("*dChip-PM/MM"*).

Like *dChip, RMA *makes use of data from all GeneChips in an experiment for normalization and considers the probe affinity effect. *RMA *uses a linear additive model and computes the expression summary values by the use of only PM probes. Normalization in *RMA *is performed by quantile normalization that transforms the PM distribution of each GeneChip in a dataset to a common distribution.

We used the three probe set algorithms to generate expression summary values for the GeneLogic dilution and diabetes datasets. For *MAS5.0*, we used the Affymetrix software suite *MAS5.0 *with its default settings and adjusted the target intensity level to 2500 to bring the total expression intensity of each GeneChip to a fixed level. We also performed further global normalization to bring the median expression values of all GeneChips to the same scale. This was done by selecting a baseline GeneChip from the dataset, followed by scaling each GeneChip to the median of the baseline GeneChip (m˜base
 MathType@MTEF@5@5@+=feaafiart1ev1aaatCvAUfKttLearuWrP9MDH5MBPbIqV92AaeXatLxBI9gBaebbnrfifHhDYfgasaacH8akY=wiFfYdH8Gipec8Eeeu0xXdbba9frFj0=OqFfea0dXdd9vqai=hGuQ8kuc9pgc9s8qqaq=dirpe0xb9q8qiLsFr0=vr0=vr0dc8meaabaqaciaacaGaaeqabaqabeGadaaakeaacuWGTbqBgaacamaaBaaaleaacqWGIbGycqWGHbqycqWGZbWCcqWGLbqzaeqaaaaa@33A4@):

xi'=m˜basem˜ixi.
 MathType@MTEF@5@5@+=feaafiart1ev1aaatCvAUfKttLearuWrP9MDH5MBPbIqV92AaeXatLxBI9gBaebbnrfifHhDYfgasaacH8akY=wiFfYdH8Gipec8Eeeu0xXdbba9frFj0=OqFfea0dXdd9vqai=hGuQ8kuc9pgc9s8qqaq=dirpe0xb9q8qiLsFr0=vr0=vr0dc8meaabaqaciaacaGaaeqabaqabeGadaaakeaacqWG4baEdaqhaaWcbaGaemyAaKgabaGaei4jaCcaaOGaeyypa0ZaaSaaaeaacuWGTbqBgaacamaaBaaaleaacqWGIbGycqWGHbqycqWGZbWCcqWGLbqzaeqaaaGcbaGafmyBa0MbaGaadaWgaaWcbaGaemyAaKgabeaaaaGccqWG4baEdaWgaaWcbaGaemyAaKgabeaakiabc6caUaaa@3F96@

For *dChip*, we used the official *dChip *version 1.3 software [27] to obtain expression summary values for both *dChip-PM *and *dChip-PM/MM*. For *RMA*, we utilized the default *rma*() function included in the "affy" package of Bioconductor in the R statistical computing environment [28]. This default function employs *median polish *for expression summary and quantile normalization for data normalization.

The expression summary values of the wholly defined control dataset were directly obtained from Choe *et al*. [14], who created 150 multiple expression summary datasets using combinations of options from probe set algorithms *MAS *(both version 4 and 5), *dChip*, and *RMA*. These options included 3 for background correction, 4 for normalization at the probe level, 3 for PM correction, 3 for expression summary, and 2 for normalization at the probe set level. In the background correction options, the use of "subtract MM for PM correction" (*MAS *version 4) resulted in negative values when PM was less than MM, and in this case about 85% of the probe sets on the GeneChip were flagged as "not applicable" when expression summaries from *MAS *and *RMA *were used. We therefore excluded 40 expression summary datasets generated from the use of this option, and employed the remaining 110 expression summary datasets in our study.

### Welch t-test

Welch *t-*tests were performed on a probe set-by-probe set basis between two sample groups; 6 × 4 for data generated from the GeneLogic dilution dataset between liver and *CNS *at the same dilution level, 1 × 4 for data generated from the diabetes dataset, and 110 for expression summary datasets from the wholly defined control dataset between two sample groups generated by the use of the same combinations of options. The Welch *t*-test is specifically designed to handle the possibility of having small samples with unequal variances, and thus has been most widely used in RNA profiling data analysis. This *t*-test relies on the assumption of data normality and homoscedansticity of expression values, which may not be valid for all data. Nevertheless, the *t*-test is the best option for our study, as it has more power than non-parametric tests such as the Wilcoxon rank sum test, in addition to its popularity for gene expression data analysis.

### Computing RPS variance based on t-statistic rank order

From each Welch *t*-test, let *n *denote the number of total null hypotheses, and *T*_*k *_the *k*-th largest *t*-statistic value, probe sets are ranked in descending order based on *t*-statistic values.

*T*_*1*_,*T*_*2*_,*T*_*3*_,*T*_*4*_, ....., *T*_*k*_, ...., *T*_*n*_.

The corresponding rank order of probe sets is

*R*_*1*_,*R*_*2*_,*R*_*3*_,*R*_*4*_, ....., *R*_*k*_, ...., *R*_*n*_,

where *R*_*1 *_= *1*, *R*_*n *_= n, *R*_*k *_the *k*-th rank order of a probe set. We compute the data variance of a gene *j *with *N*_*j *_RPSs:

σ˜j=1Nj∑n=1Nj|Rjkn−μ(Rj)|
 MathType@MTEF@5@5@+=feaafiart1ev1aaatCvAUfKttLearuWrP9MDH5MBPbIqV92AaeXatLxBI9gBaebbnrfifHhDYfgasaacH8akY=wiFfYdH8Gipec8Eeeu0xXdbba9frFj0=OqFfea0dXdd9vqai=hGuQ8kuc9pgc9s8qqaq=dirpe0xb9q8qiLsFr0=vr0=vr0dc8meaabaqaciaacaGaaeqabaqabeGadaaakeaacuaHdpWCgaacamaaBaaaleaacqWGQbGAaeqaaOGaeyypa0ZaaSaaaeaacqaIXaqmaeaacqWGobGtdaWgaaWcbaGaemOAaOgabeaaaaGcdaaeWbqaamaaemaabaGaemOuai1aaSbaaSqaaiabdQgaQjabdUgaRnaaBaaameaacqWGUbGBaeqaaaWcbeaakiabgkHiTiabeY7aTjabcIcaOiabdkfasnaaBaaaleaacqWGQbGAaeqaaOGaeiykaKcacaGLhWUaayjcSdaaleaacqWGUbGBcqGH9aqpcqaIXaqmaeaacqWGobGtdaWgaaadbaGaemOAaOgabeaaa0GaeyyeIuoaaaa@4D0E@

As an alternative, the following can also be used to compute RPS variance:

σj2=1Nj∑n=1Nj[Rjkn−μ(Rj)]2
 MathType@MTEF@5@5@+=feaafiart1ev1aaatCvAUfKttLearuWrP9MDH5MBPbIqV92AaeXatLxBI9gBaebbnrfifHhDYfgasaacH8akY=wiFfYdH8Gipec8Eeeu0xXdbba9frFj0=OqFfea0dXdd9vqai=hGuQ8kuc9pgc9s8qqaq=dirpe0xb9q8qiLsFr0=vr0=vr0dc8meaabaqaciaacaGaaeqabaqabeGadaaakeaacqaHdpWCdaqhaaWcbaGaemOAaOgabaGaeGOmaidaaOGaeyypa0ZaaSaaaeaacqaIXaqmaeaacqWGobGtdaWgaaWcbaGaemOAaOgabeaaaaGcdaaeWbqaamaadmaabaGaemOuai1aaSbaaSqaaiabdQgaQjabdUgaRnaaBaaameaacqWGUbGBaeqaaaWcbeaakiabgkHiTiabeY7aTjabcIcaOiabdkfasnaaBaaaleaacqWGQbGAaeqaaOGaeiykaKcacaGLBbGaayzxaaWaaWbaaSqabeaacqaIYaGmaaaabaGaemOBa4Maeyypa0JaeGymaedabaGaemOta40aaSbaaWqaaiabdQgaQbqabaaaniabggHiLdaaaa@4DD6@

where *k *is the *k*-th rank order for a probe set of gene *j*, and μ (*R*_*j*_) the average distance of the *N*_*j *_probe sets for gene *j*:

μ(Rj)=1Nj∑n=1NjRjkn
 MathType@MTEF@5@5@+=feaafiart1ev1aaatCvAUfKttLearuWrP9MDH5MBPbIqV92AaeXatLxBI9gBaebbnrfifHhDYfgasaacH8akY=wiFfYdH8Gipec8Eeeu0xXdbba9frFj0=OqFfea0dXdd9vqai=hGuQ8kuc9pgc9s8qqaq=dirpe0xb9q8qiLsFr0=vr0=vr0dc8meaabaqaciaacaGaaeqabaqabeGadaaakeaacqaH8oqBcqGGOaakcqWGsbGudaWgaaWcbaGaemOAaOgabeaakiabcMcaPiabg2da9maalaaabaGaeGymaedabaGaemOta40aaSbaaSqaaiabdQgaQbqabaaaaOWaaabCaeaacqWGsbGudaWgaaWcbaGaemOAaOMaem4AaS2aaSbaaWqaaiabd6gaUbqabaaaleqaaaqaaiabd6gaUjabg2da9iabigdaXaqaaiabd6eaonaaBaaameaacqWGQbGAaeqaaaqdcqGHris5aaaa@4585@

We employed *t*-statistic values to rank probe sets instead of using the *p*-values. This is because *t*-statistic values reflect the variances of RPSs in rank order more appropriately than *p*-values do, as the *t*-statistic values distinguish the up-regulation from down-regulation for RPSs, but the *p*-values do not. Another alternative for computing the variances of RPSs is to directly use the *t*-statistic values, but this approach is less robust, as *t*-statistic values from different probe set algorithms may not be in the same scale.

### Quantitative RT-PCR

Quantitative real time RT-PCR was performed for 10 genes from the diabetes dataset (Table 3). Total RNA from either normal or diabetic animals was reverse transcribed and used to generate cDNA with Invitrogen SuperScript First-Strand Synthesis System Kit (Carlsbad, CA). Primers for selected genes were designed using Primer3 developed at Whitehead Institute and Howard Hughes Medical Institute (Cambridge, MA) and synthesized by Sigma-Genosys (The Woodlands, TX). Primer sequences were designed to flank an intron near the 3' end of the gene sequence, have 20 to 22 bp, and contain 50–60% GC sequences and a G or C at the 3'end. Real time PCR was performed using cDNA, gene specific primers, SYBR Green PCR Core Reagent Kit obtained from Applied Biosystems (Foster City, CA), and the iCycler IQ Real Time PCR detection system from BioRad (Hercules, CA). As a constitutive control, the β2 microglobuin gene was used. The PCR efficiency was 98–100% for all primer sets as determined using standard curves to test linearity. The fold change of the comparison of diabetic to normal rats was calculated as 2 ^(Ct target geneD - Ct control gene)-(Ct target geneN - Ct control gene)^, where Ct is the threshold cycle.

## Abbreviations

*MAS5.0 *(Microarray Suite 5.0 statistical algorithm from Affymetrix), *dChip *or MBEI (Model based expression index), *RMA *(Robust multi-array average), PM (Perfect match probe), MM (Mismatch probe), DEG (Differentially expressed genes), RPS (Redundant probe set), qRT-PCR (Quantitative real time – polymerase chain reaction), ROC (Receiver operator characteristic).

## Authors' contributions

ZH initiated, designed, and carried out the research and drafted the manuscript. GRW supervised the preparation of the diabetes data set and PCR quantification, and prepared the diabetes dataset. All authors read and approved the final manuscript.

## Figures and Tables

**Table 1:** Number of probe sets and redundant probe sets used in two-sample *t*-tests between liver and central nervous system (*CNS*) cell lines from the GeneLogic dilution dataset.

***t*-test**	**Total # PS _a _**(filtered)	**# RPS**	**% RPS**	**# unigenes_b_**	**# unigenes for RPS**
Liver 1.25 vs. *CNS *1.25	7257	1998	27.53	5812	901
Liver 2.5 vs. *CNS *2.5	7329	2017	27.52	5871	909
Liver 5.0 vs. *CNS *5.0	7755	2224	28.68	6164	998
Liver 7.5 vs. *CNS *7.5	7905	2287	28.39	6265	1021
Liver 10.0 vs. *CNS *10.0	7456	2092	28.06	5952	940
Liver 20.0 vs. *CNS *20.0	7386	2042	27.65	5906	918

Notation: PS represents probe sets, RPS redundant probe sets. The numbers following Liver and *CNS *represent cRNA concentration.

a. The total # of probe sets in HG-U95A GeneChip is 12,627.

b. Only those with unigene labels were counted.

**Table 2:** Uniquely over-represented functional categories for DEGs obtained from each probe set algorithm from the experimental diabetes dataset.

**Functional categories _a_**	***p*-value**	**Functional categories**	***p*-value**
***MAS5.0*:**		***dChip-PM/MM*:**	
Proteolysis and peptidolysis	0.008	Negative regulation of transcription*	0.024
Ubiquitin-dependent protein catabolism	0.013	Response to stress*	0.024
Modification-dependent protein catabolism	0.015	Phosphagen biosynthesis*_b_	0.043
Intracellular protein transport	0.018	Muscle development	0.033
Intracellular signaling cascade*	0.025	Striated muscle contraction	0.043
Lipoprotein biosynthesis*	0.038	***dChip-PM*:**	
Protein lipidation	0.039	Fatty acid metabolism*	0.001
Steroid metabolism*	0.047	Organic acid metabolism	0.001
		Perception of smell	0.037
***RMA*:**		Sensory perception of chemical stimulus	0.049
Innate immune response	0.016		
Acetyl-COA metabolism	0.018		
Gutamate signaling pathway	0.034		
Inflammatory response	0.040		

* Categories known to be directly related to diabetes.

a. Biological process generated by DAVID 2.0 [21] as of Oct. 6, 2005.

b. Genes in the function category are involved in energy metabolism.

**Table 3:** Genes and primer sequences for qRT-PCR.

**Gene**	**GenBank**	**Primer sequences**	**Fold change **(diabetic vs. normal)
GSH-ST	J03914	Forward: GATGTCCTTGATCAACACCG Reverse: GGCCGCTCTTCATGTAGTCAG	5.47
MHC	x04267	Forward: GTCAAGTCCTACAAGAGGCAGG Reverse: GATTCTGCAATATCCGCACG	4.78
Metallothionein 1 and 2	M11794	Forward: CTGCGGCTGCAAGAACTG Reverse: CTTGTCCGAGGCACCTTTG	7.21
HMGCoAS	M33648	Forward: CTGCCCAAACGTCTAGACTCC Reverse: GAAGAGGTTGCTTGTGTCACC	6.2
ALS	03190	Forward: GCTTATGAGGCAGATGCTAATGG Reverse: ATGCCTGGTCATCAACTCATC	-2.96
PHAS-1	U05014	Forward: ACTAGCCCTACCAGCGATGAG Reverse: TGTCCATCTCAAACTGTGACTC	2.64
DiEcoAR	D00569	Forward: TGCAGTGATTATGCCTCTTGG Reverse: CTTCGATTACATCCCACTCCTC	2.58
CPT-1	L07736	Forward: TTGTCTACGAGCCAGACTCCTC Reverse: AGGAGACACCATAGCCGTCATC	2.42
RAD	U12187	Forward: CATCCTAGTGGGCAACAAGAG Reverse: AGGTCTCGATGAACTTGCAGTC	1.53
CK	X59736	Forward: CTGGTCGCTACTACAAGCTGTC Reverse: CCCAGCGCATGTTAGTAAAGG	-3.93
β-2-microglobulin	Y00441	Forward: CCACCGAGACCGATGTATATG Reverse: CGGATCTGGAGTTAAACTGGTC	

In the order the genes are glutathione S-transferase, embryonic skeletal muscle myosin heavy chain, metallothionein-1 and 2, mitochondrial 3-hydroxy-3methylglutaryl-CoA synthase, 5-aminolevulinate synthase, PHAS-I protein, 2,4-dienoyl-CoA reductase, carnitine palmitoyltransferase I, Ras-related protein, and sarcomeric mitochondrial creatine kinase.
